# Electro-Acupuncture Promotes Accumulation of Paclitaxel by Altering Tumor Microvasculature and Microenvironment in Breast Cancer of Mice

**DOI:** 10.3389/fonc.2019.00576

**Published:** 2019-07-02

**Authors:** Ming Yang, Yuxiang Wan, Xin Jiang, Xuewei Qi, Lina Wang, Zeyu Liu, Xiaojing Song, Lin Pan, Weiliang Sun, Wei Zhao, Jinchang Huang, Zenglin Lian

**Affiliations:** ^1^School of Traditional Chinese Medicine, Beijing University of Chinese Medicine, Beijing, China; ^2^Beijing University of Chinese Medicine Third Affiliated Hospital, Beijing, China; ^3^Department of Biomedical Engineering, Institute of Acupuncture and Moxibustion, China Academy of Chinese Medical Sciences, Beijing, China; ^4^Institute of Clinical Medical Science, China-Japan Friendship Hospital, Beijing, China; ^5^Guanganmen Hospital, Chinese Academy of Traditional Chinese Medicine, Beijing, China; ^6^Institute of Biological Chinese Medicine, Beijing Yichuang Institute of Biotechnology Industry, Beijing, China

**Keywords:** acupuncture, targeted drug delivery, paclitaxel, breast cancer, microenvironment

## Abstract

Targeted drug delivery could increase the efficacy of chemotherapy, however, a plethora of obstacles exist in the current targeted delivery designs. In this study, we introduce a novel avenue of targeted drug delivery using electro-acupuncture and evaluate its effect on the distribution of paclitaxel in a breast cancer mouse model. Our results show that electro-acupuncture intervention significantly increased the intratumoral concentration of paclitaxel. The mice in acupuncture group showed shorter *t*_max_, longer *t*_1/2_ and higher AUC of paclitaxel as compared with that in paclitaxel-only group. Moreover, we found that the acupuncture intervention significantly induced cell apoptosis in tumors. The levels of COL IV and α-SMA increased in tumors of acupuncture group. The negative tumor metastasis biomarker, NM23, was significantly upregulated in tumors of mice in acupuncture group. Our results suggest that acupuncture intervention around the tumor area increases the local concentration of chemotherapeutic agents. The targeted effect of acupuncture is achieved by altering tumor microvasculature and microenvironment. Therefore, combined therapy of acupuncture with chemotherapeutic agents is promising in improving cancer treatment efficacy.

## Introduction

The goal of targeted drug delivery system (TDDS) is to deliver a certain amount of a therapeutic agent to a targeted diseased area within the body. Compared with the conventional drug delivery system, TDDS has advantages in improving drug efficacy, reducing drug dosages, and side effects (1). In recent years, tumor-targeted drug delivery system has showed great potential in cancer therapy. However, the current TDDS has limitations, such as instability and low drug-loading capacity (2). Therefore, localizing the drug within tumors remains one of the biggest challenges in cancer chemotherapy.

Breast cancer is one of the most common malignancy in women worldwide. About 15–20% of breast cancers are found to be triple-negative (3, 4). Due to its molecular profiles, triple-negative breast cancer does not respond to hormonal therapy or therapies that target HER2 receptors. Cytotoxic chemotherapy is the major treatment choice used for triple-negative breast cancer (5). Therefore, increasing the intratumoral drug concentration is critical for improving the efficacy of chemotherapy in triple negative breast cancer. It has been reported that protein-bound paclitaxel, such as nab-paclitaxel, could increase the intratumoral concentration of paclitaxel and therefore increase treatment response compared with solvent-based paclitaxel, indicating the importance of high concentration of drug in cancer treatment (6).

Acupuncture has been used in China for thousands of years as an important part of traditional Chinese medicine (7). It has been accepted and used by at least 103 countries according to the WHO statistics (8). In recent years, acupuncture has been widely used among cancer patients as a none-drug therapy against many cancer-related symptoms including cancer-related pain, vomiting, postoperative intestinal obstruction, dry mouth, fatigue, hot flashes, anxiety, insomnia and others (9). Importantly, it has been reported that acupuncture intervention could significantly increase perfusion in local microcirculation (10), and affect the distribution of chemotherapeutic drugs in mice (11, 12). And acupuncture has also been found to play a role in the regulation of extracellular matrix (13).

In this study, we investigate whether acupuncture could navigate the chemotherapeutic drugs to reach and localize in the tumor area by changing the tumor microenvironment. We perform *in vivo* imaging and pharmacokinetic analysis to examine whether acupuncture intervention could alter the distribution of paclitaxel in triple negative breast cancer-bearing mice. We further explored whether acupuncture could change the tumor microvasculature and microenvironment in molecular level.

## Materials and Methods

### Reagents

Cy7-paclitaxel was purchased from FanBo Biochemicals Co. Ltd. (Beijing, China). Injecting paclitaxel is the product of SL Pharm Co. Ltd. (Beijing, China). Monoclonal antibodies against COL I (ab34710), COL IV (ab6586), CD31 (ab28364), CD34 (ab81289), α-SMA (ab5694), HIF1A (ab2185), VEGFA (ab1316), and NM23A (ab154547) were acquired from Abcam (Cambridge, MA) and were used in immunohistochemistry and Western blot assays.

### Establishment of 4T1 Breast Cancer Mouse Models

Female BALB/C nude mice were used for *in vivo* imaging and female BALB/C mice were used for pharmacokinetic analysis, immunohistochemical studies and Western blot analysis. All the animals were purchased from Beijing Vital River Laboratory Animal Technology Co., Ltd. (Beijing, China) and maintained at China-Japan Friendship Hospital Animal Facility with access to water and rodent chow *ad libitum*. The breast cancer 4T1 cells (1 × 10^6^) were suspended in 0.1 mL PBS and injected into the 4th left inguinal mammary gland fat pad (14, 15). Models were considered successfully established when tumors became palpable (100–300 mm^3^) (16). All animal procedures were conducted according to the guidelines and approval of the Animal Care & Welfare Committee of China-Japan Friendship Hospital (approval No. 171102).

### Electro-Acupuncture Intervention

Sterile acupuncture needle (size 0.18 × 15 mm, Suzhou DongBang Medical Co., Ltd., Suzhou, China) was used to perform electro-acupuncture interventions. Specifically, 4 needles were inserted toward the tumor at up, down, left and right directions in tumor-bearing mice, or the 4th left mammary gland fat pad in normal mice under anesthesia. The site of acupuncture is around 5 mm off the boundary of tumor in tumor-bearing mice or mammary fat pad in control mice and the acupuncture depth is around 5 mm. The electro-acupuncture interventions were performed by SDZ-II electronic acupuncture treatment device (Hwato Co., Suzhou, China) using longitudinal wave setting (low frequency: 3–4 Hz, 5 s; high frequency: 15–20 Hz, 10 s). The representative acupuncture intervention setup in mice is shown in Supplementary Figure S1.

### *In vivo* Fluorescence Imaging

The BALB/c nude mice were randomized into 4 groups, normal group (NG), electro-acupuncture normal group (EANG), tumor group (TG), and electro-acupuncture tumor group (EATG), with 7 mice in each group. After the establishment of 4T1 breast cancer models in mice in TG and EATG groups, all mice were intravenously injected with 0.2 mL cy7-paclitaxel (0.1 mg/mL) under anesthesia (17, 18). X-ray can clearly show the prone position for the fluorescence imaging (Supplementary Figure S2). Electro-acupuncture interventions were performed as a single treatment in EANG and EATG group for 20 min after cy7-paclitaxel injection. Fluorescence images and X-ray images were captured in FX-RPO *in vivo* imaging system (Carestream Health Inc., Rochester, NY) at the time point immediately after the injection, and 0.33, 2.33, 4.33, 6.33, 24.33, and 48.33 h after cy7-paclitaxel injection. The instrumental parameters were set as follows: excitation wavelength, 720 nm; emission wavelength, 790 nm; exposure time, 10 s, f-Stop = 2.50, FOV = 120.0 mm, binning = 2.

### Pharmacokinetic Analysis

The BALB/c mice were randomized into 4 groups as described in 2.4 with 8 mice in each group at each time points. After the establishment of 4T1 breast cancer models in TG and EATG groups, all mice were intravenously injected with a single dosage of paclitaxel (10 mg/kg) under anesthesia. Electro-acupuncture interventions were performed as a single treatment in EANG and EATG groups for 20 min after paclitaxel injection. Ten time points were designed for blood collection at 0.033, 0.083, 0.167, 0.333, 0.833, 1.333, 2.333, 4.333, 6.333, and 24.333 h. Tissue samples, including heart, liver, spleen, lung, kidney, tumor (TG and EATG group), and skin from acupuncture area or non-acupuncture area (NG and EANG group), were excised at 0.033, 0.833, 2.333, 4.333, 6.333, and 24.333 h and stored at −80°C until used. The paclitaxel concentrations in tissue samples were measured by Agilent 1,100 LC-MS/MS (Agilent Technologies, Inc., Santa Clara, CA). The experimental design was based on several studies (19–21) and took into considerations of the results of fluorescence imaging. Besides, we have professional advices from ZLA (Beijing) Pharmaceutical Technology Co., Ltd.

### Immunohistochemistry and TUNEL Assay

The BALB/c mice were randomized into 5 groups, normal group (NG), tumor group (TG), paclitaxel tumor group (PTG), electro-acupuncture tumor group (EATG), and electro-acupuncture combined with paclitaxel tumor group (EAPTG), with 6 mice in each group. After the establishment of 4T1 breast cancer models in all groups except NG group as previously described, the mice received a single dosage of 10 mg/kg paclitaxel i.v., injection under anesthesia. Electro-acupuncture interventions were performed as a single treatment in EATG and EAPTG group after paclitaxel injection. The mice were sacrificed 2 h after paclitaxel injection and the tumors were excised for immunohistochemistry and TUNEL assays. Immunohistochemistry assays were performed following the established DAB IHC protocol, using the following primary antibodies: anti-COL I (1:200, ab34710, Abcam. UK), anti-COL IV (1:200, ab6586, Abcam. UK), anti-CD31 (1:50, ab28364, Abcam. UK), anti-CD34 (1:200, ab81289, Abcam. UK), anti-alpha SMA (1:200, ab5694, Abcam. UK), anti-HIF1A (1:200, ab2185, Abcam. UK), anti- VEGFA (1:100, ab1316, Abcam. UK), or anti-NM23A (1:100, ab154547, Abcam. UK). Slices with normal IgG as a primary antibody were used as a negative control. Cell apoptosis in tumors was detected by TUNEL analysis using the *in situ* Cell Death Detection Kit, POD (Roche, Basel, Switzerland) following the manufacturer's instruction.

All mice (each group consists of 6 mice) were included in the IHC analyses. The immunohistochemical analyses were performed on 5 μm-thick paraffin sections. Four views were randomly chosen from each specimen to be imaged by Image-Pro PLUS v6.0 (Media Cybernetics Inc., Rockville, MD). Both immunohistochemistry and TUNEL results were assessed by H-score system (22, 23). The formula for the H-score is: H-SCORE = ∑(PI × I) = (percentage of cells of weak intensity × 1) + (percentage of cells of moderate intensity × 2) + percentage of cells of strong intensity × 3), where I = intensity of staining and Pi = percentage of stained tumor cells. Each experiment was repeated three times.

### Western Blot Analysis

Tumor tissues from the 5 groups of mice as described in section Immunohistochemistry and TUNEL assay were used for Western blot analysis. The tissues were lysed by RIPA buffer and the protein concentrations were measured by BCA assays. Previously established Western blot protocol was followed (22, 23). The signals were quantified by AlphaEase software (Genetic Technologies, Inc., Miami, FL).

### Data Processing and Statistical Analysis

The *in vivo* imaging results were analyzed using Bruker MI SE software (Bruker Corp. Billerica, MA). The non-compartmental pharmacokinetic analysis was performed using WinNonlin v6.3 (Certara Inc., Princeton, NJ). The time-averaged relative drug exposure (Re) and drug targeting efficacy (Te) were calculated as previously described by Gupta et al. (24). $\text{Re}=\left(\text{AU}{\text{C}}_{\text{tissue}}\text{EATG}\right)/\left(\text{AU}{\text{C}}_{\text{tissue}}\text{TG}\right),\text{Te}=\text{AU}{\text{C}}_{\text{tissue}}/\overset{\text{n}}{\underset{\left(\text{j}=1\right)}{\sum }}{\left(\text{AUC}\right)}_{\text{j}}\left(\text{j refers to each tissue}\right).$ The statistical differences were determined by independent sample *t*-test, one-way ANOVA or Mann-Whitney u-test using SPSS v22. Differences were considered significant when *p* < 0.05.

## Results

### *In vivo* Fluorescence Imaging and Pharmacokinetic Analysis

**Figure 1:**
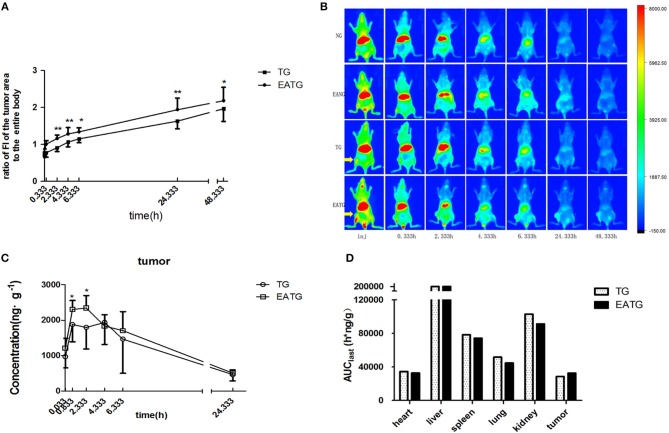
Electro-acupuncture intervention increased paclitaxel accumulation in tumor area. **(A)** The ratio of fluorescence intensities in tumor area to the entire body in TG and EATG group after cy7-paclitaxel injection, inj: the time point immediately after injection, ^*^P < 0.05, ^**^P < 0.01 (mean ± SD, *n* = 7). **(B)** Visualization of cy7-PTX-*in vivo* fluorescence imaging among the groups (*n* = 7). **(C)** The intratumoral paclitaxel concentration-time curves in TG and EATG group after paclitaxel injection, ^*^P < 0.05 (mean ± SD, *n* = 8). **(D)** The AUC among organs and tumor. The *p*-values were calculated using the student's *t*-test or Mann-Whitney *U* test in **(A,C)**. The AUC was calculated with the average concentration at each time point in different mouse so the statistical significance is not applicable in Figure 1D.

In order to evaluate the distribution of paclitaxel in different groups of mice, we first performed *in vivo* fluorescence imaging and pharmacokinetic analysis after intravenous injection of cy7-paclitaxel. As shown in Figure 1A, the fluorescence intensity of the tumor area to the entire body is initially similar between TG group (0.708 ± 0.070) and EATG group (0.781 ± 0.093) after paclitaxel injection (*p* = 0.121). However, the fluorescence intensity in tumor area to the entire body of EATG group was significantly higher than those in TG group from 20 min to 24.333 h after paclitaxel injection. Representative *in vivo* fluorescence images are shown in Figure 1B.

We further measured the pharmacokinetic parameters in different groups. Consistent with the imaging results of tumor sites, the intratumoral paclitaxel concentration was significantly higher in EATG group as compared to TG group at time points of 0.833 h (*p* = 0.046) and 2.333 h (*p* = 0.028) (Figure 1C). The acupuncture intervention in EATG group decreased the t_max_ and increased the peak concentration of paclitaxel inside tumors and the AUC (area under curve) of tumor was higher in EATG group (Supplementary Table S1; Figure 1D). There were no significant difference of paclitaxel distribution in the skin between ipsilateral electro-acupuncture group and non-electro-acupuncture group (Supplementary Figure S3A). For the acupuncture site (left) or the contralateral site (right), there was no difference between two sides of skin in terms of paclitaxel concentration in NG group, in which the animal did not receive acupuncture intervention (Supplementary Figure S3B). However, the acupuncture intervention in EANG group significantly increased the concentration of paclitaxel in acupuncture site at 2.333 h (*p* = 0.048) and 4.333 h (*p* = 0.021) (Supplementary Figure S3C). What's more, there were no significant differences in paclitaxel pharmacokinetic parameters in lung, liver, spleen, kidney, and heart between TG group and EATG group (Supplementary Table S1; Supplementary Figure S4). Further analysis (Table 1) showed that the acupuncture increased the tumor targeted efficacy of paclitaxel [Te(EATG)/Te(TG) = 1.185]. These results suggested the improved concentration from other non-targeted organs to the tumor with the electro-acupuncture intervention.

**Table 1:** Tumor-targeting abilities of paclitaxel in the EATG and TG group (*n* = 8).

**Tissues**	**Heart**	**Liver**	**Spleen**	**Lung**	**Kidney**	**Tumor**
Re^a^	0.948	1.014	0.948	0.864	0.886	1.143
Te^b^(TG)	0.069	0.404	0.158	0.104	0.208	0.057
Te(EATG)	0.068	0.425	0.155	0.093	0.191	0.068
Te(EATG)/Te(TG)	0.984	1.052	0.983	0.896	0.919	1.185

a *Re: intake ratio*.

b *Te: targeting efficacy*.

*The time-averaged relative drug exposure (Re) and drug targeting efficacy (Te) were calculated as previously described by Gupta et al. (24)*.

Besides, the paclitaxel concentration in plasma was notably increased in EATG group at 0.333 h compared to TG group (*p* = 0.037) and EANG group at 0.333 h (*p* = 0.011) and 4.333 h (*p* = 0.015) compared to NG group. Concentration-time curves and pharmacokinetic parameters are shown in Supplementary Figure S5; Supplementary Table S2.

### Acupuncture Combines With Paclitaxel Induces Tumor Cell Apoptosis

**Figure 2:**
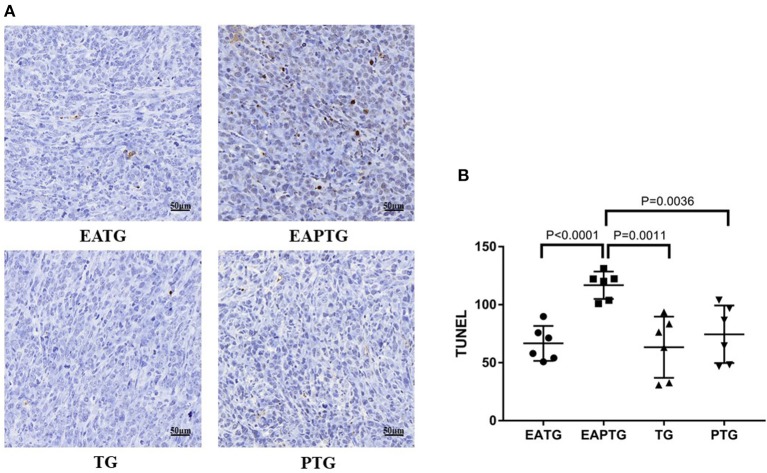
Acupuncture combined with paclitaxel treatment promoted cell apoptosis in 4T1 xenograft tumors. **(A)** TUNEL staining on representative tumor sections from the EATG, EAPTG, TG, and PTG groups. 400 ×. **(B)** Quantitative comparison of TUNEL staining among these four groups (one-way ANOVA test; mean ± SD, *n* = 6).

We further evaluate the antitumor effect paclitaxel injection in the present or absent of electro-acupuncture intervention using TUNEL assays. Tumor samples were collected 2 h after paclitaxel injection. As shown in Figure 2, there was no significant difference on TUNEL apoptosis index between electro-acupuncture alone EATG group, paclitaxel alone PTG group and no treatment control TG group. However, acupuncture and paclitaxel combined EAPTG group had a significantly elevated level of cell apoptosis. These results indicated that combined acupuncture intervention and paclitaxel treatment significantly induces apoptosis in 4T1 breast cancer cells *in vivo*.

### Acupuncture Stimulates Microvasculature and Microenvironment

In order to understand the mechanism of acupuncture intervention induced accumulation of paclitaxel in the tumor site, we evaluated expression of molecules related to tumor microvasculature and microenvironment.

**Figure 3:**
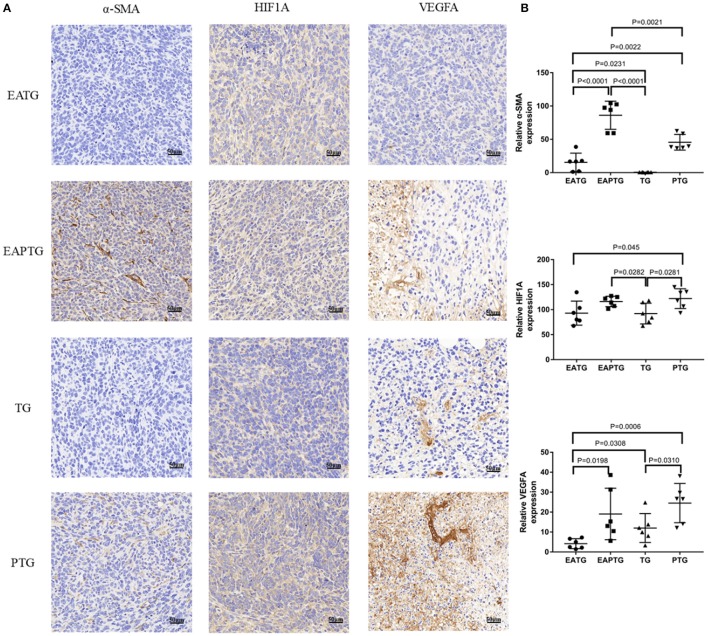
Immunohistochemical evaluation in 4T1 xenograft tumors treated with electro-acupuncture combined with paclitaxel. **(A)** Representative tumor sections from the EATG, EAPTG, TG, and PTG group. The sections are stained with α-SMA, HIF1A, and VEGFA. 400 ×. **(B)** Quantitative comparison of H-scores of α-SMA, HIF1A, and VEGFA in these four groups (one-way ANOVA test; mean ± SD, *n* = 6).

Unlike blood vessels in normal tissues, tumor vasculature has abnormal structure and function, forming a physiological barrier to the delivery of therapeutic agents to tumors. We first stained the tumor tissues with vascular endothelial biomarkers CD31 and CD34; we found the expression of CD31 was not significantly different among these groups. There was no significance in the expression of CD34 either, which may merely due to the two outliers in TG group (Supplementary Figure S6). In addition to vascular endothelial cells, activated carcinoma-associated fibroblast in peritumoral tissues plays an essential role in tumor microvasculature. As shown in Figure 3, a significantly higher level of α-smooth-muscle actin (α-SMA) was observed in EAPTG group than in other groups. In addition, the hypoxic tumor microenvironment is subjected to HIF-driven transcriptional responses in tumor cells. The HIF-1-VEGF signaling pathway can be activated and promoted neovascularization. Therefore, we stained and compared HIF1A and VEGFA in these tumor tissues. HIF1A level was significantly upregulated in EAPTG group and PTG group as compared to TG group and downregulated in EATG group as compared to PTG group (*p* = 0.045). There was no significant difference on HIF1A level among EAPTG group and PTG group. We also found that compared to TG group, VEGFA level was upregulated in PTG group and downregulated in EATG group. However, there was no statistical difference.

**Figure 4:**
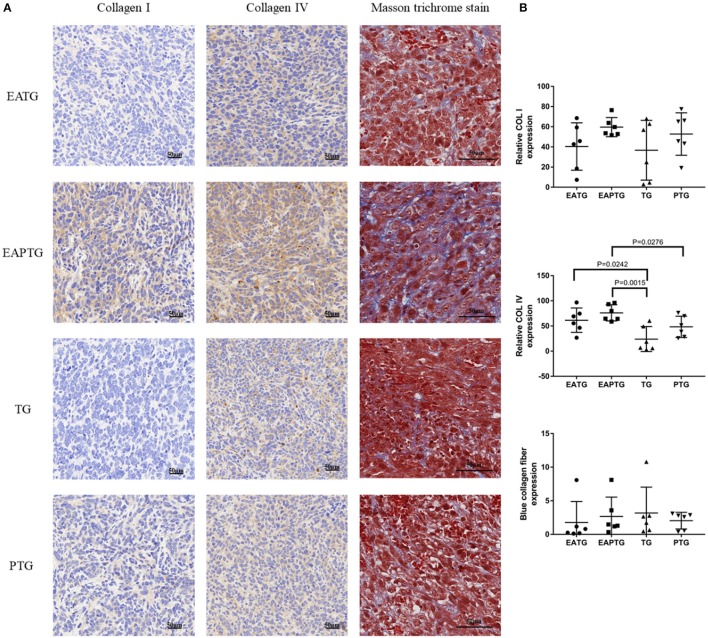
Immunohistochemical evaluation on the extracellular matrix in 4T1 xenograft tumors treated with electro-acupuncture combined with paclitaxel. **(A)** Representative tumor sections from the EATG, EAPTG, TG, and PTG group. The sections are stained with COL I, COL IV or with Masson trichrome stain. 400 ×. **(B)** Quantitative comparison of H-scores of COL I, COL IV, and blue collagen fiber area in Masson trichrome stain in these four groups (one-way ANOVA test; mean ± SD, *n* = 6).

Collagens are the major component of the extracellular matrix (ECM). The mechanical stress caused by tumor cells and nearby collagens could decrease the blood flow in tumor vasculature. In order to evaluate whether acupuncture could interfere with collagen synthesis in ECM, we performed Masson's trichrome staining and IHC staining for COL I and COL IV (Figure 4). We found the proportion of collagens stained blue was not significantly different among these groups in Masson's trichrome staining. On the other hand, The IHC results showed that COL I were not significantly changed among different groups. Interestingly, we found that COL IV was significantly upregulated in acupuncture-only EATG group and paclitaxel combined acupuncture EAPTG group as compared to TG group. Besides, the COL IV level in EAPTG group was notably higher than that in PTG group. Moreover, morphology analysis showed that the COL IV in EAPTG group was intact and distributed in a linear pattern. These results indicate that acupuncture regulates the change of microvasculature and microenvironment.

### Acupuncture on Tumor Metastasis

**Figure 5:**
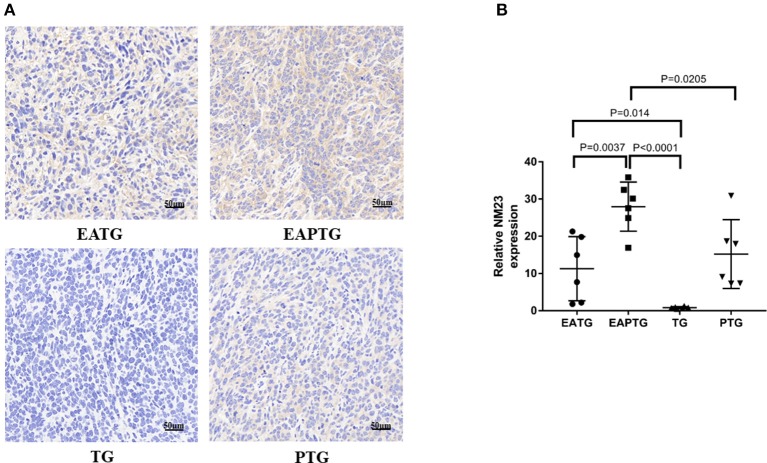
Immunohistochemical evaluation on the invasion and metastasis of 4T1 xenograft tumors treated with electro-acupuncture combined with paclitaxel. **(A)** IHC staining using NM23 antibody on tumor sections from the EATG, EAPTG, TG, and PTG group. 400 ×. **(B)** Quantitative comparison of H-score of NM23 in these four groups (one-way ANOVA test; mean ± SD, *n* = 6).

We also evaluated the safety of acupuncture in tumor area in terms of tumor invasion and metastases. The H&E staining of lung and liver sections were used for evaluating metastatic tumors. No lung metastasis was detected and there is no significant difference in liver metastasis among these 4 groups (Supplementary Figure S7). We examined the expression of NM23, a tumor metastasis suppressor, using immunohistochemical staining. It was found that the expression of NM23 in EAPTG group and EATG group were significantly up-regulated as compared with TG group. The expression of NM23 was significantly higher in EAPTG group than those in PTG group (Figure 5). These results indicate that acupuncture does not promote tumor metastasis.

### Western Blot Analysis

**Figure 6:**
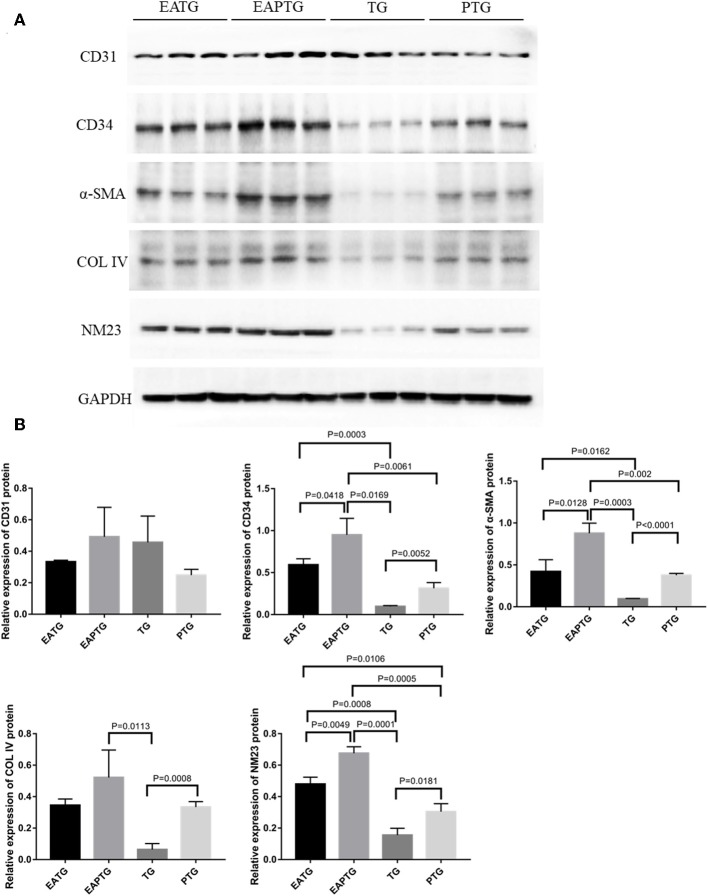
Western blot analysis of key targets in 4T1 xenograft tumors. **(A)** Representative blots of CD31, CD34, α-SMA, COL IV, NM23, and loading control GAPDH in EATG, EAPTG, TG, and PTG group. **(B)** Densitometric analysis of the immunoblots using the AlphaEase software (one-way ANOVA test; mean ± SD, *n* = 3).

In order to further validate those aforementioned biomarkers, we performed Western blot analysis using the tumor tissues from different treatment groups (Figure 6). Consistent with IHC results, we found that there is no significant difference in CD31 levels among these 4 groups. EAPTG group exhibited a significantly higher expression of CD34 and α-SMA as compared to TG group or PTG group. Moreover, EAPTG group also exhibited a higher level of COL IV as compared to TG group or PTG group. However, the difference of COL IV between EAPTG group and PTG group did not reach statistical significance. Finally, the significantly higher level of NM23 in EAPTG group as compared with TG group or PTG group was also validated by Western blot analysis.

## Discussion

We started this study to explore whether electro-acupuncture could increase drug accumulation within tumor tissues. We found that the fluorescence intensity within tumor area was higher in electro-acupuncture group than that in control group after cy7-paclitaxel injection in several time points. This result suggested that electro-acupuncture could increase the concentration of chemotherapeutic agents in the tumor area. We profiled the pharmacokinetic parameters of paclitaxel following intravenous injection in the present or absent of electro-acupuncture intervention. The results confirmed that acupuncture could increase the intratumoral drug concentration. In a previous study, Peng found that acupuncture can influence the action of Tf-Cy7 targeting and distribution of Dox (11). Zhang's study showed increased paclitaxel concentration in organs after acupuncture, and multi-needle method was better than single-needle for the targeting effects (12). We also found that acupuncture increased the skin drug concentration at acupuncture site. These results suggest that the effect of acupuncture induced drug accumulation may not be tumor-specific. Besides, the elevated TUNEL apoptosis index in paclitaxel combined acupuncture group further suggested that acupuncture intervention may lead to increased paclitaxel cytotoxicity most likely by increasing the intratumoral drug concentration.

It has been reported that acupuncture could increase blood circulation in affected areas. Heterogeneous perfusion exists within tumors might hinder the delivery of drugs to poorly perfused areas (25). The increased accumulation of chemotherapeutic agent in tumor after the intervention might be related to the change of microvasculature induced by acupuncture. We evaluated several biomarkers related to tumor microvasculature. We selected endothelial biomarkers CD31 (26) and CD34 (27) as the biomarkers for the total amount of blood vessels and the amount of mature differentiated blood vessel. Our results show that acupuncture did not promote proliferation of the total number of blood vessels, but increased the number of differentiated functional blood vessels within tumor microvasculature. In addition to endothelial cells, the pericytes are also essential for maintaining normal microvasculature (28). It has been reported that insufficient pericytes are correlated with incomplete blood vessel structures in tumors. The low pericyte coverage is related to severe tumor invasion and poor prognostics in breast cancer patients (29). In this study, we examined the level of α-SMA to evaluate the alternations of pericytes. We found significantly higher level of α-SMA in paclitaxel combined acupuncture group as compared to paclitaxel-only group. This result indicated that acupuncture may facilitate the perfusion within tumors by increasing the pericytes and normalizing the tumor microvasculature without alter the total number of blood vessels.

It is interested to find that there was no difference of HIF1A level between EATG group and TG group, but a considerable higher level of HIF1A was observed in EAPTG group and PTG group compared to TG group. Besides, the VEGFA expression was downregulated in EATG group, and upregulated in PTG group compared to TG group. These results suggest that acupuncture could alter the hypoxic microenvironment and affect the process of angiogenesis in tumors. However, HIF1A level was higher in EAPTG group than in TG group and VEGFA level was not different between the two groups. Because the tumor samples were acquired at a single time point, more study is needed to understand the related mechanism.

ECM plays important roles in drug delivery (30). Rapid growing tumor mass can deform tumor surrounding tissues and compress intratumoral blood vessels. Compression of blood vessels reduces blood flow and in turn lowers the efficacy of chemotherapeutics (31, 32). A previous report showed that acupuncture may induce ECM remodeling (33). We evaluated the effects of acupuncture on several key components of ECM. The result of Masson's trichrome staining revealed that acupuncture combined with paclitaxel did not facilitate ECM proliferation at the time point of 2 h. We analyzed the expression of type I collagen (COL I) as the marker of mesenchyme and type IV collagen (COL IV) as the marker of basement membrane in the tumor tissue (34). It was interested to find that the expression of COL I remained unchanged, but the expression of COL IV increased in EAPTG group. These results indicated that acupuncture did not affect the proliferation of mesenchymal cells, but improved the completeness of basement membrane.

There were concerns whether acupuncture could promote tumor metastasis. We evaluated metastatic loci on lung and liver sections and found that acupuncture did not promote tumor invasion and metastasis of 4T1 breast cancer cells. NM23 is a gene identified by Steeg et al. which is associated with tumor metastasis (35). It was reported that the NM23 protein level was high in normal cells and tumors of low metastatic potential (36, 37). We found that NM23 level in tumor tissue was significantly higher in acupuncture EAPTG group, suggesting that electro-acupuncture therapy combined with paclitaxel are unlikely to increase the metastasis risk of 4T1 cancer cells. This result was in consistent with the above mentioned high level of COL IV and α-SMA in EAPTG group, because the integrity of microvascular basement membrane reduces the risk of metastasis (38, 39).

In conclusion, electro-acupuncture combined with paclitaxel therapy could increase the drug concentration in the 4T1 breast cancer xenografts by regulating the microvasculature and microenvironment of the tumor. However, this study merely focused on the effect after 2 h of the electro-acupuncture intervention, more researches are needed to further investigate the effects of continuous interventions and other drug combinations. Nevertheless, it is encouraging to apply electro-acupuncture with chemotherapy for cancer treatment.

## Data Availability

No datasets were generated or analyzed for this study.

## Ethics Statement

All animal procedures were conducted according to the guidelines and approval of the Animal Care & Welfare Committee of China-Japan Friendship Hospital (approval No. 171102).

## Author Contributions

JH and ZenL: conceived and designed the study. MY and YW: performed experiments and analyzed the results. XJ, XQ, LW, ZeyL, XS, LP, WS, and WZ: performed parts of experiments and analyzed results. MY and YW: wrote the first draft. JH and ZenL: edited the manuscript. All authors read and approved the manuscript.

### Conflict of Interest Statement

The authors declare that the research was conducted in the absence of any commercial or financial relationships that could be construed as a potential conflict of interest.
