# Supplementary material for: Electro-Acupuncture Promotes Accumulation of Paclitaxel by Altering Tumor Microvasculature and Microenvironment in Breast Cancer of Mice
Source: Front Oncol. 2019 Jul 2;9:576. doi: 10.3389/fonc.2019.00576 (PMC6614178; doi:10.3389/fonc.2019.00576)
Supplement: Supplementary file 1 [file Data_Sheet_1.docx]

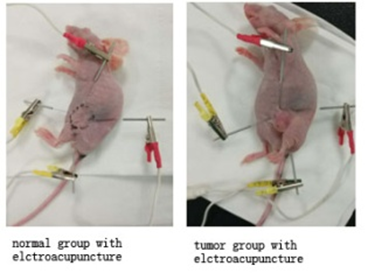


**Supplementary Figure S1**. The setup of acupuncture in normal and tumor-bearing mice.


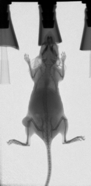


**Supplementary Figure S2.** X-ray shows the prone position of a mouse for fluorescence imaging of the tumor.


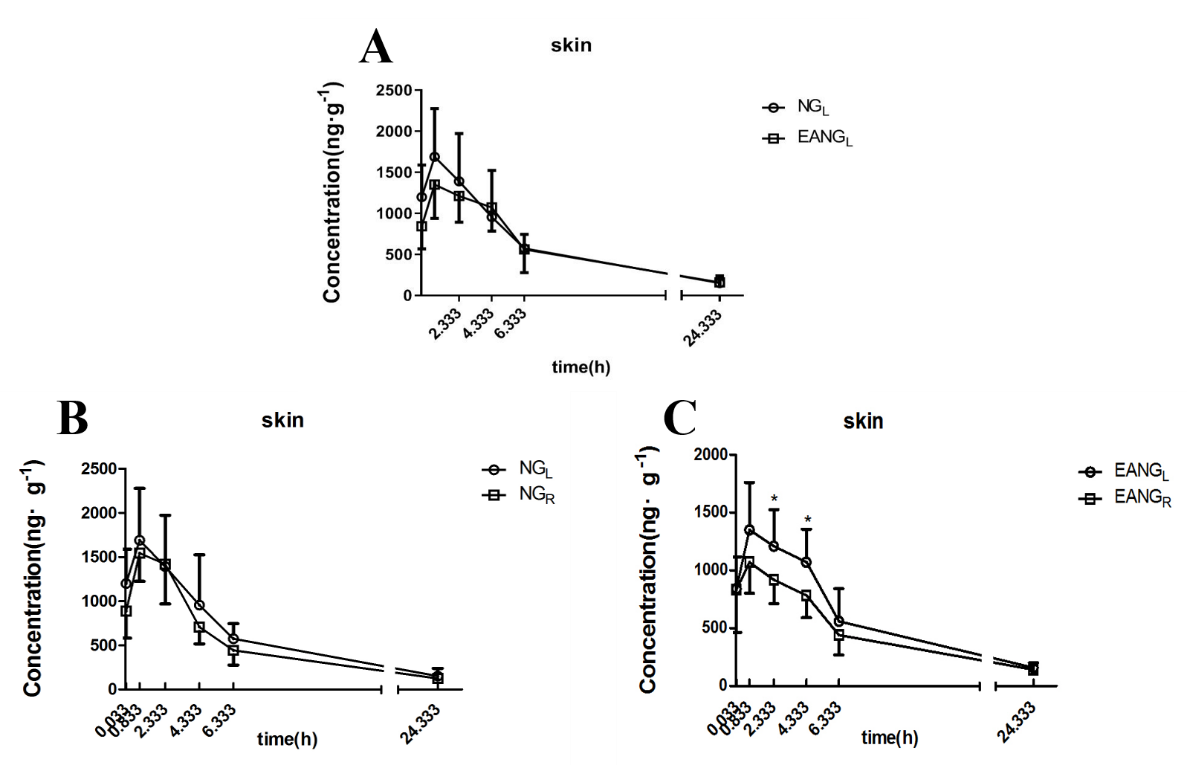


**Supplementary Figure S3** The concentration of paclitaxel in the skin of mice in EANG group and NG group. (A) ipsilateral skin in EANG group and NG group. (B) the paclitaxel concentration-time curves of left and right skin in NG group. (C) the paclitaxel concentration-time curves in left (acupuncture site) and right (the contralateral site) skin in EANG group. The p values were calculated using student’s t-test or Mann-Whitney U test. *p < 0.05 (mean±SD, n=8).


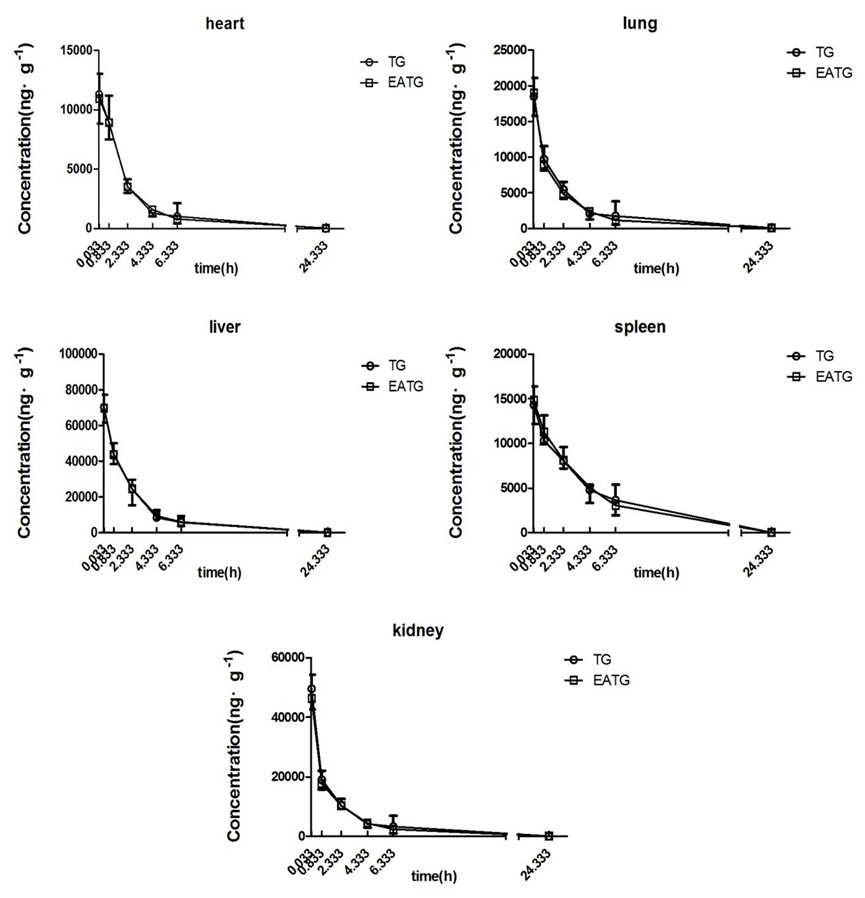


**Supplementary Figure S4**. The concentration-time profiles of paclitaxel in heart, lung, liver, spleen and kidney in TG and EATG group. The p values were calculated using student’s t-test or Mann-Whitney U test. *p < 0.05 (mean±SD, n=8).


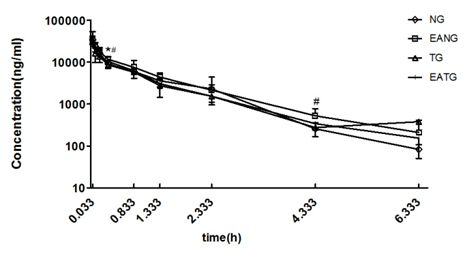


**Supplementary Figure S5** Plasma concentration-time curves of NG, EANG, TG, EATG after i.v. administration to Balb/c female mice at the same 10mg/kg PTX dose. The p values were calculated using student’s t-test or Mann-Whitney U test in the comparison of TG&EATG or NG&EANG.TG&EATG: *P<0.05, NG&EANG: #P<0.05) (mean±SD, n=8)


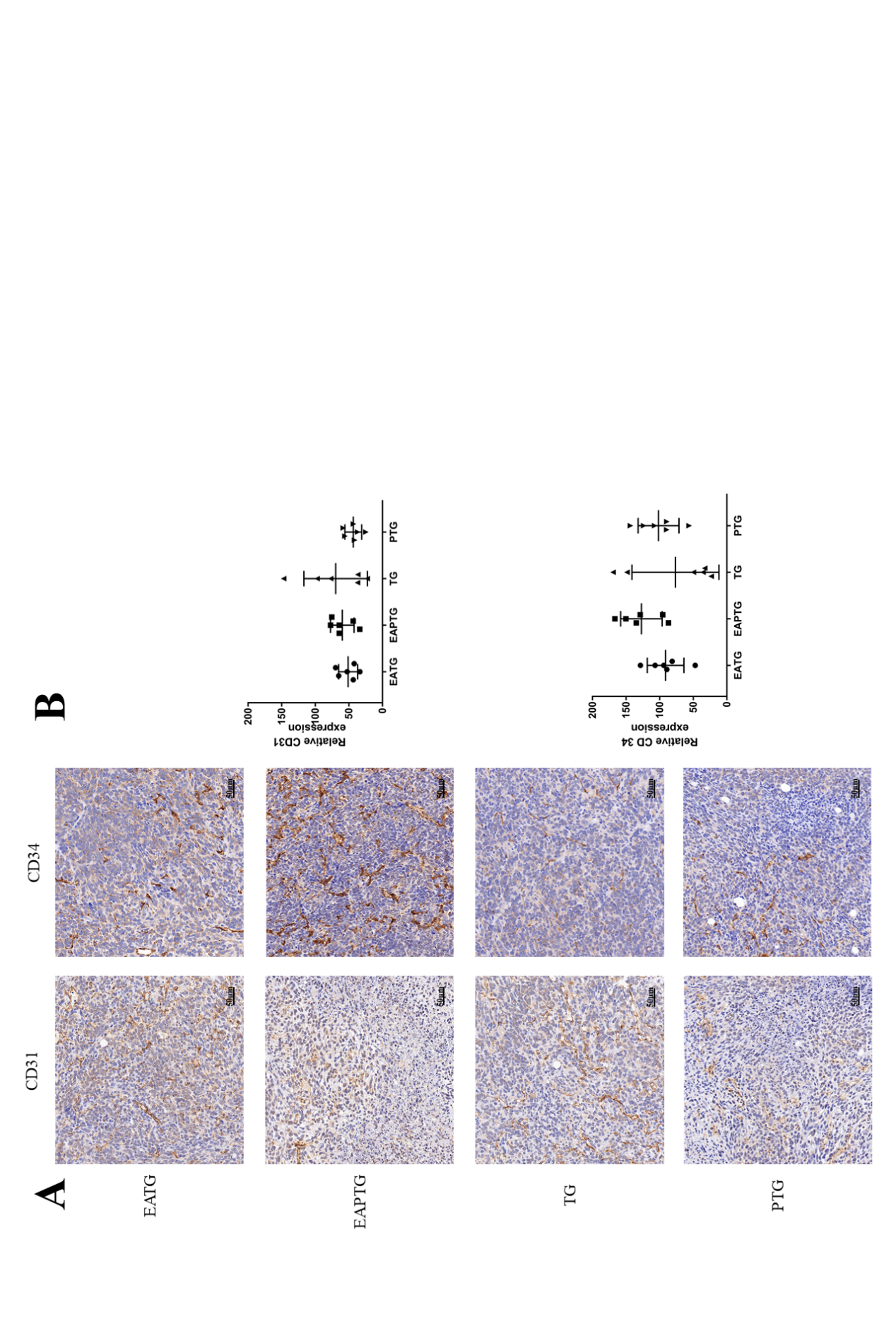


**Supplementary Figure S6**. Immunohistochemical evaluation of the effect of acupuncture on the intratumoral vasculature. (A) Immuno-staining of CD31 and CD34 in EATG, EAPTG, TG and PTG group. (B) Quantitative comparison of H-score of CD31 and CD34 from the four groups. (one-way ANOVA test; mean±SD, n=6)


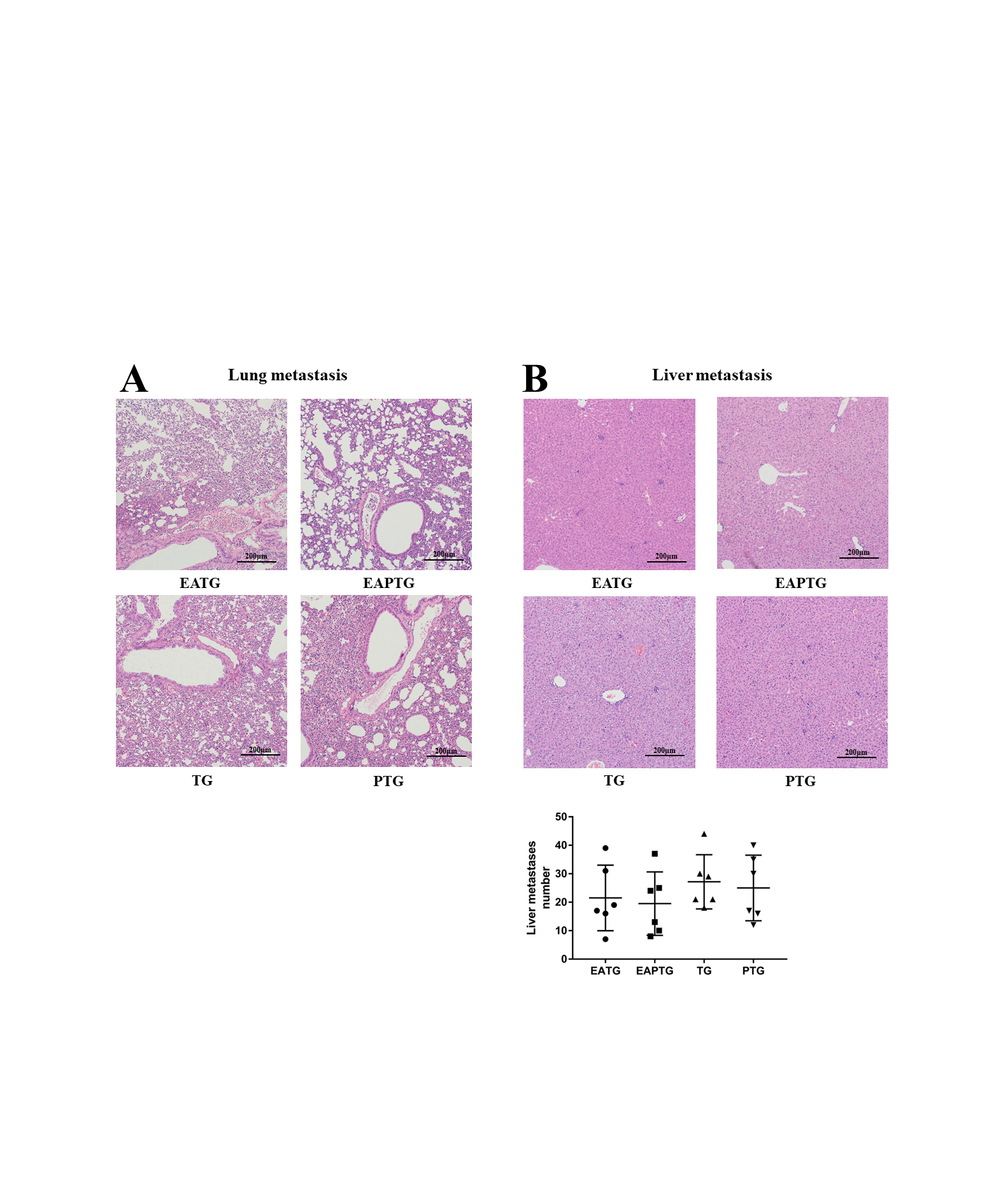


**Supplementary Figure S7**. Histological evaluation of the effect of acupuncture combined with paclitaxel on invasion and metastasis. (A) Representative examples for the lung sections from the EATG, EAPTG, TG and PTG groups. (B) Representative examples for the liver sections from the EATG, EAPTG, TG and PTG groups and the quantitative comparison of the number of metastasis. All H&E staining sections are shown in 100×(one-way ANOVA test; mean±SD, n=6).

**Supplementary Table S1**. The pharmacokinetic parameters in tumor and tissues after intravenous injection of the paclitaxel in mice. The non-compartmental pharmacokinetic analysis was performed using WinNonlin v6.3 (Certara Inc., Princeton, NJ) (n=8).

|  | Heart | | Liver | | Spleen | |
| --- | --- | --- | --- | --- | --- | --- |
|  | TG | EATG | TG | EATG | TG | EATG |
| t_1/2_ (h) | 2.806 | 3.954 | 3.337 | 3.316 | 2.351 | 2.353 |
| T_max_ (h) | 0.033 | 0.033 | 0.033 | 0.033 | 0.033 | 0.033 |
| C_max_ (ng/g) | 11291.645 | 10948.288 | 70132.867 | 69678.913 | 14285.414 | 14811.911 |
| AUC_last_ (h*ng/g) | 34371.662 | 32601.016 | 200293.06 | 203139.2 | 78173.299 | 74121.92 |

|  | lung | | Kidney | | Tumor | |
| --- | --- | --- | --- | --- | --- | --- |
|  | TG | EATG | TG | EATG | TG | EATG |
| t_1/2_ (h) | 4.402 | 4.547 | 2.838 | 2.908 | 10.162 | 10.591 |
| T_max_ (h) | 0.033 | 0.033 | 0.033 | 0.033 | 4.333 | 2.333 |
| C_max_ (ng/g) | 18507.766 | 19044.576 | 49527.782 | 46296.143 | 1938.47 | 2342.931 |
| AUC_last_ (h*ng/g) | 51588.66 | 44579.328 | 102934.98 | 91191.401 | 28506.508 | 32570.697 |

**Supplementary Table S2**. The pharmacokinetic parameters in plasma after intravenous injection of the paclitaxel in mice The non-compartmental pharmacokinetic analysis was performed using WinNonlin v6.3 (Certara Inc., Princeton, NJ) (n=8).

|  | NG | EANG | TG | EATG |
| --- | --- | --- | --- | --- |
| t_1/2_ (h) | 0.863 | 1.125 | 1.071 | 1.139 |
| T_max_ (h) | 0.333 | 0.333 | 0.033 | 0.033 |
| C_max_ (ng/g) | 42957.6 | 47978.66 | 52398.5 | 46207.42 |
| AUC_last_ (h*ng/g) | 18621.42 | 21802.13 | 16111.24 | 17167.78 |
| MRTlast(h) | 0.993 | 1.052 | 1.038 | 0.965 |
